# The distinct link of perfectionism with positive and negative mental health outcomes

**DOI:** 10.3389/fpsyt.2025.1492466

**Published:** 2025-03-14

**Authors:** Patricia D. Simon, Maria Guadalupe C. Salanga, John Jamir Benzon R. Aruta

**Affiliations:** ^1^ Department of Education Studies, Hong Kong Baptist University, Hong Kong, Hong Kong SAR, China; ^2^ Department of Psychology, De La Salle University – Manila, Manila, Philippines

**Keywords:** perfectionism, high standards, discrepancy, positive mental health, negative mental health, Filipino undergraduate students, well-being, bidimensional mental health

## Abstract

Perfectionism is a known risk for mental health symptoms. However, the literature on perfectionism and mental health mostly focused on the psychopathological symptoms when mental health is not only about the absence of psychopathology but also the presence of positive indicators. The present study aimed to examine the associations of adaptive and maladaptive dimensions of perfectionism with bidimensional mental health among undergraduate students (*N* = 467) at a private university in the Philippines. We assessed the role of High Standards and Discrepancy perfectionism on the negative (depression, anxiety, and stress) and positive (satisfaction with life and meaning in life) indicators of mental health. Structural equation modeling demonstrated distinct associations of High Standards and Discrepancy on mental health and well-being outcomes. High Standards positively predicted stress and life satisfaction, and negatively predicted depression, presence of meaning, and search for meaning. High Standards did not have a significant effect on anxiety. Discrepancy positively predicted depression, anxiety, and stress and negatively predicted life satisfaction. Interestingly, presence of meaning was significantly and positively associated with Discrepancy while search for meaning was not. This study contributes to the literature by finding evidence for the distinct influence of Discrepancy and High Standards on several indicators of positive and negative mental health.

## 1 Introduction

Perfectionism is characterized by a tendency to set excessively high performance standards for the self and a propensity to engage in negative self-evaluations (1, 2). Certain personality traits could make people susceptible to mental health issues. One such trait is perfectionism (3, 4). Perfectionism is a transdiagnostic personality construct that shares a relationship with various mental health outcomes (5). However, the literature on perfectionism and mental health mostly focused on the psychopathological symptoms (6) when mental health is not only about the absence of psychopathology but also the presence of positive indicators (7). Therefore, the objective of the present study is to examine the adaptive and maladaptive dimensions of perfectionism and their relationships with negative (depression, anxiety, stress) and positive indicators (satisfaction in life and meaning in life) of mental health. Doing so can potentially provide a balanced perspective to the dearth of studies on perfectionism and mental health. Below, we provide support for the study’s objectives.

These tendencies may lead to negative mental health outcomes. Specifically, perfectionism has been linked to depressive symptoms [e.g., (8, 9)] and is a risk factor for major depressive disorder (10). Other negative outcomes associated with perfectionism are psychosomatic distress (11), psychological distress (12, 13), athlete burnout (14), posttraumatic stress disorder symptoms (15), and various forms of psychopathology and emotional dysfunction (6). However, researchers also found evidence for the adaptiveness of perfectionism, and thus acknowledge that the trait has both adaptive and maladaptive aspects (16).

Various terms are used to define adaptive and maladaptive perfectionism. For the present study, we employed the conceptualization of perfectionism of Slaney and colleagues (17): High Standards, Discrepancy, and Order. High Standards captures the tendency to set extremely high standards of excellence for oneself. Discrepancy is the perception of failing to meet the standards and expectations one has set for the self. Order centers on being disciplined and organized. The Order dimension of perfectionism is often excluded in studies about perfectionism since researchers tend to place more emphasis on the more problematic aspects of perfectionism (High Standards and Discrepancy) that are more strongly linked to psychological distress and maladaptive outcomes (18). Given this trend in perfectionism research to study dimensions with clear implications for mental health interventions (19), we adopted the same approach in the current study.

Slaney et al.’s model that includes High Standards and Discrepancy (2001) captures perfectionism’s adaptive and maladaptive aspects and have different consequences and associations with mental health outcomes. Discrepancy as measured by the Almost Perfect Scale-Revised [APS-R; (17)] shares similarities with perfectionism constructs that are viewed as maladaptive such as evaluative concerns (1) and socially-prescribed perfectionism (20). Discrepancy is also related with negative mental health outcomes such as psychological distress, depression, and anxiety symptoms (21). High Standards as measured by the APS-R (17) shares similarities with perfectionism constructs that are considered to be adaptive such as personal standards (1) and self-oriented perfectionism (20). High Standards is also associated with positive mental health outcomes and was found to be negatively related to depression (22). In another study, students who scored high on APS-R’s subscale High Standards had higher mean overall scores in psychological well-being and its subscales: positive relations with others, environmental mastery, autonomy, and purpose in life, relative to those who scored high in Discrepancy (23). These studies suggest that perfectionism, although considered as a pathological trait due to its numerous links to negative psychological outcomes, also has a positive impact on mental health.

In terms of the bidimensionality of mental health, Westerhof and Keyes (7) described the traditional conceptualization of mental health as focusing on the absence or presence of psychopathology and proposed that the conceptualization of mental health must extend by taking into account both the positive (well-being and flourishing indicators) and negative aspects (psychopathological symptoms) of mental health or bidimensional mental health. The World Health Organization (24) recognizes this conceptualization by characterizing a mentally healthy individual as an individual who not only copes with daily life’s stresses but also experiences a sense of satisfaction, fulfillment, and contribution to society. The literature on perfectionism predominantly focuses either on the negative or positive mental health [e.g., (25, 26)]. Expanding the examination of perfectionism and negative mental health by incorporating positive indicators of mental health is a step forward in recognizing that mental health is a bidimensional construct (27). In this study, we selected satisfaction with life and meaning in life (presence of meaning and search for meaning) as indicators of positive mental health and depression, anxiety, and stress symptoms as indicators of negative mental health.

We examined the adaptive and maladaptive dimensions of perfectionism and its consequences on positive (satisfaction with life, meaning in life) and negative indicators (depression, anxiety, and stress) of mental health among Filipino undergraduate students. Previous studies present inconsistent findings on the link between perfectionism and mental health constructs. There are studies that outline the adaptive aspects of perfectionism and its positive effects on mental health (28). On the other hand, several studies also revealed strong associations between perfectionism and negative mental health outcomes (5, 6) such as depressive symptoms [e.g., (10, 29)], eating pathology (30), and anxiety (5, 31). In one study, both perfectionism subscales showed a positive relationship with hopelessness and suicidal ideation (32). Additionally, High Standards, although often considered as the more adaptive dimension of perfectionism, also demonstrates an ambivalent relationship with well-being outcomes, at times showing significant association with both positive and negative aspects of well-being (33). These studies bring to view concerns about perfectionism dimensions’ relationship with positive and negative indicators of mental health. Importantly, there has been a consistent upsurge in perfectionism among university students for three decades now based on a meta-analysis (34).

Culturally, interdependent cultures like the Philippines are known to have a unique conception and predictors of mental health and well-being (35) and that models and findings dominated by Western samples often do not operate similarly when tested among non-Western samples (36). For example, findings based on Filipino samples revealed that self-criticism, often known in Western literature to be detrimental to mental health, was found to have adaptive functions against depression and anxiety among Filipinos with high levels of interdependent self-construal (37). In terms of the relationship between perfectionism and bidimensional mental health, there remains a dearth of studies that focus on their relationship in the context of non-Western populations, especially the Philippines. The present study, therefore, recruited university students in the Philippines.

Below, we present all of our hypotheses:

1. High standards will be positively associated with positive mental health (life satisfaction, presence of meaning, and search for meaning) and negatively associated with negative mental health (depression, anxiety, and stress).
2. Discrepancy will be positively associated with negative mental health (depression, anxiety, and stress) and negatively associated with positive mental health (life satisfaction, presence of meaning, and search for meaning).

## 2 Materials and methods

### 2.1 Participants and procedure

Data were gathered from undergraduate students (*N* = 467) from a private university in the Philippines through an online survey. Among these, 304 (65%) were females, and 159 (34%) were males. Four respondents chose not to disclose their gender. Respondents were on average 19.72 years old (*SD* = 1.29). No power analysis was performed prior to gathering data for the study.

Online survey links with the informed consent form were sent to school administrators, faculty, and directly to the email inboxes of students through the university’s learning management system. The respondents were also invited to participate through social media pages of student organizations. Data collection was conducted from the last week of June 2021 to the second week of July 2021 during the peak of the COVID-19 pandemic. Apart from four respondents who refused to disclose their gender, participants completed all items in the survey and no missing data were found. Additionally, no outliers were identified. The study adhered to ethical standards set by the American Psychological Association and the study protocol was reviewed and approved by the Velez College Ethics Review Committee, with protocol code VCERC-2021-NON-020.

### 2.2 Instruments

#### 2.2.1 Perfectionism

The Almost Perfect Scale-Revised [APS-R; (17)] was used to measure perfectionism. It has 23-items composed of three subscales: High Standards (7 items; e.g. “I have high expectations for myself”), Discrepancy (12 items; e.g. “Doing my best never seems to be enough”), and Order (4 items; e.g. “I am an orderly person”). Only the Discrepancy and High Standards subscales were measured in the current study, as studies have consistently shown that these perfectionist dimensions are more predictive of mental health concerns (18, 38). The High Standards subscale captures the degree of striving one has for high personal performance (adaptive perfectionism) while the Discrepancy subscale measures the degree of distress a person experiences due to the perceived gap between one’s performance and standards (maladaptive perfectionism). Respondents were asked to choose in the range of a 7-point Likert scale (1=strongly disagree to 7=strongly agree). In this study, all of the subscales of the APS-R displayed adequate Cronbach’s alpha reliability (.86; High standards,.93; Discrepancy; Order;.75).

#### 2.2.2 Depression, anxiety, and stress

Depression, Anxiety and Stress Scale-21 [DASS-21; (39)] was utilized in this study to measure depression, anxiety, and stress symptoms. It is a short version of DASS-42 composed of three 7-item self-report subscales that measure depression (7 items; e.g. “I felt that I had nothing to look forward to”), anxiety (7 items; e.g. “I felt scared without any good reason”), and stress (7 items; e.g. “I found it difficult to relax”). Responses to items ranged from 0 = *did not apply to me at all* to 3 = *applied to me very much, or most of the time*, depending on the degree to which the respondents concur with the statements. The scores for each subscale are added and multiplied to two for equivalence with DASS-42. In the current study, internal consistencies based on Cronbach’s alpha for the depression, anxiety, and stress subscales were.89,.85, and.83, respectively.

#### 2.2.3 Satisfaction with life

The five-item Satisfaction with Life Scale [SWLS; (40)] was used to measure life satisfaction. The scale intends to capture an individual’s general positive attitude toward one’s life. Sample item is “I am satisfied with my life.” Respondents were asked to rate each item from 1 (strongly disagree) to 7 (strongly agree). The SWLS is a commonly used measure of well-being. In the present study, it displayed adequate Cronbach’s alpha reliability at.80.

#### 2.2.4 Meaning in life

Meaning in Life was assessed using the Meaning in Life Questionnaire [MLQ; (41)]. MLQ measures the extent to which respondents feel their lives are meaningful (MLQ-presence of meaning subscale; 5 items, e.g. “I have a good sense of what makes my life meaningful”) and also the extent to which they are actively seeking meaning in their lives (MLQ-search for meaning, subscale; 5 items, e.g. “I am seeking a purpose or mission for my life”). Each meaning dimension was measured by a Likert scale which ranges from 1 (absolutely untrue) to 7 (absolutely true). The internal consistency of the presence of meaning subscale in the current study was.52, while the search for meaning subscale demonstrated a.93 reliability based on Cronbach’s alpha.

## 3 Results

### 3.1 Descriptive statistics and correlations

The descriptive statistics, correlations among the variables and reliability of the scales employed are presented in **Table 1**.

**Table 1:** Descriptive statistics, scale reliability, and correlations among variables.

Variable	*M*	*SD*	α	1	2	3	4	5	6	7	8
1. HS	39.84	6.97	.86	--	.27**	.01	.16**	.26**	.08	-.21**	-.21**
2. Disc	55.90	15.64	.93		--	.53**	.43**	.51**	-.33**	-.11*	.21**
3. Dep	16.52	10.96	.89			--	.64**	.69**	-.49**	-.04	.33**
4. Anx	17.33	10.79	.85				--	.78**	-.28**	-.11*	.14**
5. Str	20.31	9.99	.83					--	-.33**	-.08	.20**
6. SWL	19.99	6.01	.80						--	.09	-.33**
7. SFM	14.06	7.89	.93							--	.25**
8. POM	19.22	5.18	.52								--

*N* = 467; *M*, mean; *SD*, standard deviation; *α*, Cronbach alpha; HS, High Standards; Disc, Discrepancy; Dep, Depression; Anx, Anxiety; Str, Stress; SWL, Satisfaction with Life; SFM, Search for Meaning; POM, Presence of Meaning. **Correlation is significant at p <.01; *Correlation is significant at p <.05.

High Standards was positively and significantly correlated with anxiety and stress, and negatively and significantly correlated with search for meaning and presence of meaning. High Standards were not significantly correlated with depression and subjective well-being. Discrepancy was positively and significantly correlated with depression, anxiety, stress, and presence of meaning, and negatively and significantly correlated with subjective well-being and search for meaning.

### 3.2 Structural equation modeling

We used a combination of absolute and incremental fit indices to investigate the goodness of fit of the data. The cut-off includes Comparative fit index (CFI) and Tucker Lewis index (TLI) values ranging from.90 and.95, and root mean square error approximation (RMSEA) and standardized root mean square residual (SRMR) below.08 are considered to indicate adequate fit. Meanwhile, TLI and CFI above.95, and RMSEA and SRMR below.05 suggest a good model fit (42). The SEM performed to examine the relationships between High Standards, Discrepancy, and the positive and negative indicators of mental health indicated adequate fit for some indicators, but resulted in less than adequate in others: χ2 (1402) = 3148.20, *p* <.001; χ2/df = 2.25; CFI = .89, TLI = .88, RMSEA = .05, 90% CI: [.05,.05; SRMR = .06]. In this model, the two perfectionism dimensions (High Standards and Discrepancy) and all of the mental health outcomes were allowed to covary.

After modification indices check, correlated error terms of the indicators of the same latent factors were also permitted to covary, resulting in an improved fit: χ2 (1364) = 2450.07, *p* <.001; χ2/df = 1.80; CFI = .93, TLI = .92, RMSEA = .04, 90% CI: [.04,.04; SRMR = .06].

**Figure 1:**
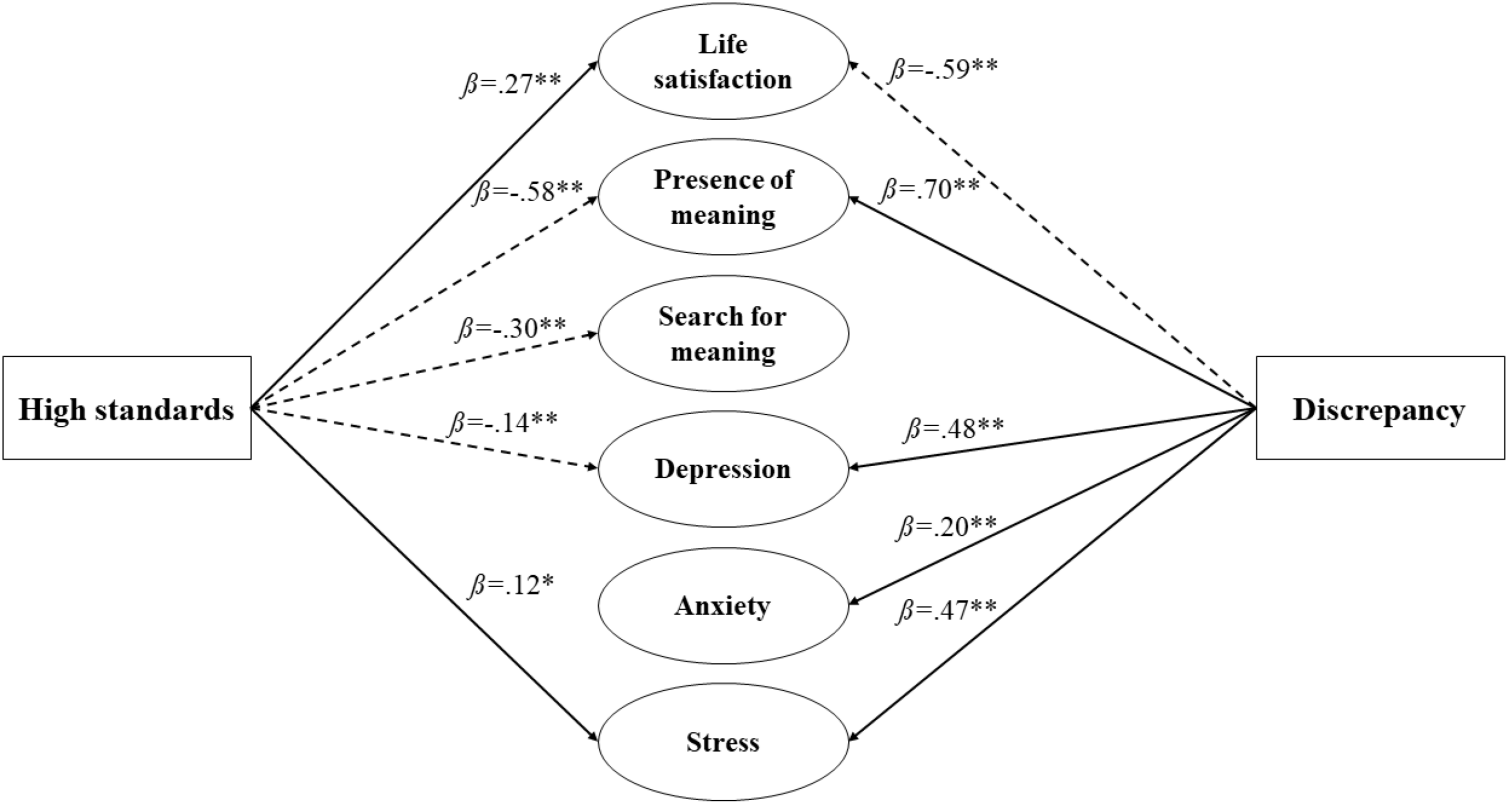
Final path analysis model representing perfectionism domains and their influence on positive and negative indicators of mental health. Solid lines represent significant and positive regression paths while dashed lines represent significant and negative regression paths **p<.001, *p<.01.

As shown in **Figure 1**, High Standards positively predicted stress and life satisfaction, and negatively predicted depression, presence of meaning, and search for meaning. High Standards’ effect on anxiety was non-significant. Discrepancy, on the other hand, positively predicted all indicators of negative mental health (depression, anxiety, and stress) and negatively predicted life satisfaction. While Discrepancy is positively associated with presence of meaning, its effect on search for meaning was non-significant. SPSS version 28.0 was used to analyze the data.

## 4 Discussion

The objective of the present study was to examine the association of the two dimensions of perfectionism (High Standards and Discrepancy) with bidimensional mental health, which includes positive (life satisfaction, presence of meaning, and search for meaning) and negative aspects (depression, anxiety, and stress). Overall, we found partial support for our hypotheses on the relationship of High Standards and Discrepancy with both positive and negative aspects of mental health. We detail our findings in the succeeding sections.

### 4.1 Perfectionism and positive indicators of mental health

We found partial support for H1 as High Standards positively predicted life satisfaction. In other words, individuals who set certain high performance standards for themselves tend to feel more fulfilled in their life in general. By setting high performance standards, it is possible that individuals are able to attain important life goals that give them happiness and fulfillment. This finding confirmed past studies which showed that setting high standards for oneself was associated with higher levels of life satisfaction [e.g., (43, 44)], as High Standards was found to positively predict selected domains of life satisfaction. Contrary to H1 and previous research (44), we found an opposite pattern as High Standards negatively influenced both presence of meaning and search for meaning in the Philippine sample. It is possible that people who set high standards for themselves develop a narrow vision of their goals, leading to frustrations and excessive preoccupation with achieving the goals that they fail to realize that it is also the journey toward achieving their goals that provides meaning in the experience. Another explanation is that life experiences appear to be less meaningful when individuals set unrealistic standards, which often results in excessive self-criticism—a known predisposing factor for poor psychological health (45).

In H2, we predicted that Discrepancy will negatively predict the three indicators of positive mental health including life satisfaction, presence of meaning, and search for meaning. Our findings showed that Discrepancy negatively influenced life satisfaction, as hypothesized. Contrary to the study’s expectation, Discrepancy positively predicted presence of meaning and did not predict search for meaning. Overall, our findings only partially confirmed H2. Previous studies supported the present findings as Discrepancy negatively predicted life satisfaction [e.g., (43, 46)]. As people with higher levels of Discrepancy perfectionism highlight the gap between their actual performance and the unrealistic standards that they set for themselves (47), it is not surprising that the participants with high Discrepancy perfectionism also reported lower levels of life satisfaction.

Contrary to H2 and previous research (26, 44), we found that Discrepancy was positively correlated with presence of meaning. Interestingly, this finding seems to suggest that people who perceive a wide gap between their standards and actual performance may consider their performance not as a failure but as a source of meaning and purpose. Existential perspectives in psychology pointed out that failures and dissatisfaction with life outcomes do not always lead to detrimental mental health and well-being consequences, especially when one could find purpose and meaning from the experience of failure (48, 49). In support of this notion, we further examined our results to look for other indicators that presence of meaning could be active during low points in people’s lives. Indeed, our results indicate a positive correlation between presence of meaning and depression symptoms. This is consistent with the existential view of negative emotions as not necessarily pathological but rather as a signal of something meaningful happening in one’s life. The positive relationship between Discrepancy and presence of meaning, and presence of meaning and depression symptoms, suggest that people may derive purpose when they process unfortunate events in their lives. For instance, the narratives and popular personal accounts of Viktor Frankl (50) highlighted his sufferings in the German concentration camps during the World War where he found profound meaning in his life after he lost everything including his family. This could explain why people with high Discrepancy tend to report greater levels of presence of meaning in life. However, the present research found no significant association between Discrepancy and search for meaning. It is possible that people with higher levels of Discrepancy perfectionism already have a strong sense of meaning in attaining their goals and performance standards, and thus need less searching for their own purpose. As indicated above, our findings revealed that Discrepancy was positively associated with presence of meaning. We note that the reliability of participants’ score in meaning in life was relatively low at Cronbach’s alpha.52, while the reliability of meaning in life based on the original study (41) ranged from.81 and.91. It is possible that the sources and indicators of meaning in life in Filipinos do not exactly reflect the conceptualization of Steger and colleagues (41) whose findings were based on undergraduate students in the U.S. enrolled in introductory psychology classes. Further research is needed to explore the conceptualization and sources of meaning in life in Filipinos.

### 4.2 Perfectionism and negative indicators of mental health

In H1, we hypothesized that High Standards will negatively influence the indicators of negative mental health including depression (e.g., persistent depressed mood, feelings of hopelessness, worthlessness, helplessness, loss of interest, etc.), anxiety (e.g., restlessness, fatigue, difficulty concentrating, irritability, palpitations, etc.), and stress symptoms (e.g., feeling overwhelmed, being emotional than usual, feeling fatigued, and trouble making a decision, solving problems, etc.). We only found partial support for H1 as High Standards significantly and negatively predicted depression symptoms but positively predicted stress symptoms and did not significantly influence symptoms of anxiety. Consistent with the present findings, previous evidence showed that High Standards was negatively correlated with depression in undergraduate populations [e.g., (51, 52)]. Setting high standards for oneself could be an indication of positive core beliefs about one’s capacity and potential (e.g., *“I am competent and capable of achieving my goals.”*), which is a known protective factor against depressive symptoms (53). On the other hand, our findings showed that High Standards positively predicted stress, which only partially supported H1 and previous research (54). This goes to show that while people who set high standards are less likely to experience depressive symptoms, they may not be free from the stresses caused by the demands of the high standards that they set for themselves as high standards often require difficult tasks and challenging obstacles.

Moreover, our findings elucidated that High Standards did not significantly predict anxiety. Results of past research among Canadian and Chinese university students (55) were consistent with the present finding. Other studies also showed that High Standards was uncorrelated with other domain-specific anxiety such as test anxiety and social anxiety symptoms among adolescents and adults [e.g., (56, 57)]. Other factors can be considered to explain this lack of correlation. For instance, a previous study on Filipino undergraduate students found that personal standards perfectionism (a construct considered synonymous with High Standards) did not lead to anxiety when parent autonomy support was high (31). Furthermore, Nguyen and Deci (56) found that susceptibility to control, or ability to control their behavior, could interact with personal standards perfectionism in predicting anxiety. In the case of the present study, it appears that High Standards was more closely linked with depression and stress symptoms than anxiety.

H2 predicted that Discrepancy will positively influence depression, anxiety, and stress. Confirming H2, the present findings revealed that Discrepancy significantly and positively predicted depression, anxiety, and stress symptoms. In other words, people who perceive a wide discrepancy between their standards and performance are more prone to developing symptoms of depression (e.g., persistent depressed mood, feelings of hopelessness, worthlessness, and helplessness, loss of interest, etc.), anxiety (e.g., restlessness, fatigue, difficulty concentrating, irritability, palpitations, etc.), and stress (e.g., feeling overwhelmed, being emotional than usual, feeling fatigued, and trouble making a decision, solving problems, etc.). Past studies supported these findings. For instance, Gnilka et al. (46) demonstrated that Discrepancy was positively correlated with hopelessness (a known symptom of depression).

Additionally, higher scores in Discrepancy were positively associated with greater symptoms of anxiety [e.g., (47, 52)] and domain-specific anxiety such as test anxiety [e.g., (58, 59)]. Furthermore, Discrepancy was found to positively predict stress symptoms (54), including among adolescents with alcohol-related problems [e.g., (60)]. Realizing the wide gap between one’s performance standards and actual performance may lead people with high Discrepancy perfectionism to engage in negative evaluations of their core self-beliefs (e.g., *“I am incompetent.”, “I am not good enough.”*), which are known predisposing and perpetuating factors for depression, anxiety, and stress symptoms.

There are also cultural implications and insights offered by the current findings. Patterns of findings on perfectionism found in the West are similar to patterns found in Filipino samples (61). Similar to established findings on perfectionism from Western samples [e.g., (8, 10)], findings from perfectionism studies in the Philippines suggest that patterns of negative psychological outcomes are all too common for perfectionists (62). Perfectionistic tendencies that are focused on evaluative concerns have an impact on academic engagement (63). Students with perfectionistic tendencies are pressured to appear that they are capable of exceptional and flawless performance and in instances when they see that their performance falls below par, they feel a sense of shame (62).

Taken together, the findings of the present research contributed to the literature by determining the influence of the two dimensions of perfectionism on both positive and negative indicators of mental health (7). The current research demonstrated the distinct influence of Discrepancy and High Standards perfectionism on several indicators of positive (life satisfaction, presence of meaning, and search for meaning) and negative indicators (depression, anxiety, and stress symptoms) of mental health. We were also able to present evidence for how a maladaptive form of perfectionism (Discrepancy) could spur processing of meaningful events in one’s life by showing the positive association between Discrepancy and presence of meaning. We were likewise able to find evidence that presence of meaning could incite negative emotions through demonstrating the positive relationship between presence of meaning and depressive symptoms. This was unexpected as a past study revealed that individuals who fit in profiles with high presence of meaning have more adaptive functioning than those with low presence of meaning (64). The positive association between presence of meaning and depressive symptoms suggests that when people have meaning in their lives, it does not absolve them from feeling negative emotions. This explanation is aligned with WHO’s (24) definition of well-being as being more than the absence of psychopathology. Importantly, the current findings offer important insights for clinical practice by illustrating that the mental health outcomes of perfectionism can go beyond the pathological symptoms or negative indicators of mental health such as depression, anxiety, and stress. Clinical practitioners may also consider the distinct link of perfectionism with the well-being aspect or positive indicators of mental health such as satisfaction with life and meaning in life.

### 4.3 Limitations and future directions

The current study has a few caveats that can serve as directions for future research. No power analysis was done *a priori*. to confidently detect that significant results were found. Future studies are recommended to conduct power analysis and other important statistical analyses to ensure robustness of the findings. The study had university student samples. Younger samples (i.e. undergraduate students) may not be representative of the larger population (65). Younger people may still be actively crafting their sense of self and identity, and thus may not have had as much time and experiences to reflect on and clarify their sources of meaning and life satisfaction compared to older people (65). It is recommended that future research examine the associations between perfectionism dimensions and bidimensional mental health with an older population. It is important to note that the data were collected during the COVID-19 pandemic but no measures to statistically control for COVID-19-related variables were employed. Moreover, diversity in sampling can also factor in socioeconomic-related variances. Future research may consider looking into socioeconomic factors in examining the influence of achievement-related traits and behaviors on a range of mental health outcomes. Specifically, the role that economic advantage plays may need to be further examined given its association with achievement-related traits such as perfectionism. In a sample of Norwegian students, perfectionism was positively associated with perceived economic well-being (66). Students from advantaged backgrounds may come from families where there is emphasis on behaviors characteristically associated with perfectionism such as goal setting and high achievement orientation.

## Data Availability

The raw data can be found in OSF: https://osf.io/6hvms.
